# Idiopathic granulomatous mastitis with GMENA syndrome: clinical features, glucocorticoid response, and prognosis – a 12-year retrospective cohort study from a single center

**DOI:** 10.3389/fimmu.2026.1883473

**Published:** 2026-08-05

**Authors:** Pin Wang, Quanling Ou, Ling Li, Ying Liu, Jian Wu

**Affiliations:** 1 Department of General Surgery, The Third People’s Hospital of Chengdu, Chengdu, China; 2 Center of Breast and Thyroid Surgery, The Third People’s Hospital of Chengdu, Chengdu, China; 3 Department of Pathology, The Third People’s Hospital of Chengdu, Chengdu, China; 4 Department of Ultrasound, The Third People’s Hospital of Chengdu, Chengdu, China

**Keywords:** erythema nodosum, glucocorticoids, GMENA syndrome, idiopathic granulomatous mastitis, recurrence

## Abstract

**Background:**

GMENA syndrome (granulomatous mastitis, erythema nodosum, with or without arthritis syndrome) is a rare subtype of idiopathic granulomatous mastitis (IGM). However, large cohort studies systematically evaluating its clinical features, response to glucocorticoid therapy, and prognosis are lacking.

**Methods:**

This single-center retrospective cohort study included 201 patients diagnosed with IGM at the Third People’s Hospital of Chengdu between January 2014 and January 2026. Among them, 20 patients presented with GMENA syndrome (GMENA group) and the remaining 181 served as the control group. Demographic characteristics, lesion features, laboratory findings, and treatment outcomes were collected for both groups. The two groups were compared regarding glucocorticoid treatment response (early improvement and total treatment duration) and recurrence rate.

**Results:**

Compared with the control group, GMENA patients were younger at onset (31–40 years: 80.0% vs. 49.7%, *p* = 0.01) and had a shorter interval from symptom onset to presentation (85.0% vs. 23.8% presenting within 9 days, *p* < 0.01). They also exhibited more extensive disease: multiquadrant involvement (100.0% vs. 14.4%), mass size > 3 cm (100.0% vs. 74.0%), multifocal lesions (100.0% vs. 18.8%), and subcutaneous abscess (60.0% vs. 15.5%) (all *p* < 0.01). All GMENA patients had unilateral involvement. The GMENA group showed a early improvement to glucocorticoids (significant improvement within 1 week: 75.0% vs. 34.8%, *p* < 0.01), but required a longer treatment duration (>6 months: 65.0% vs. 8.8%, *p* < 0.01) and had a significantly lower recurrence rate (5.0% vs. 33.7%, *p* < 0.01). Autoantibody panels were negative in both groups, and no significant difference was found in pus culture positivity rates.

**Conclusion:**

GMENA syndrome is a distinct clinical subtype of IGM characterized by acute onset, more severe breast involvement, rapid initial response to glucocorticoids, and, after an adequate prolonged course, favorable long-term outcomes with low recurrence. Early recognition and a treatment strategy of prompt initiation followed by sufficient consolidation are recommended to avoid unnecessary surgery.

## 1 Introduction

Idiopathic granulomatous mastitis (IGM) is a rare chronic inflammatory breast disease of unknown etiology, histopathologically defined by non-caseating granulomas centered on the breast lobules. Since its first description in 1972, the condition has drawn continuous attention from both breast surgeons and rheumatologists due to its complex pathophysiology and heterogeneous clinical presentations (1, 2). IGM typically affects women of childbearing age with a history of childbirth. Clinically, it presents as a painful breast mass, skin erythema, abscess formation, and sinus tract development. Radiologically, IGM often mimics breast cancer, and core needle biopsy is required for definitive diagnosis (3). Although there is no standardized treatment protocol for IGM, glucocorticoids remain the most used first-line therapy, as recommended by the international multidisciplinary consensus (4).

**Figure 1:**
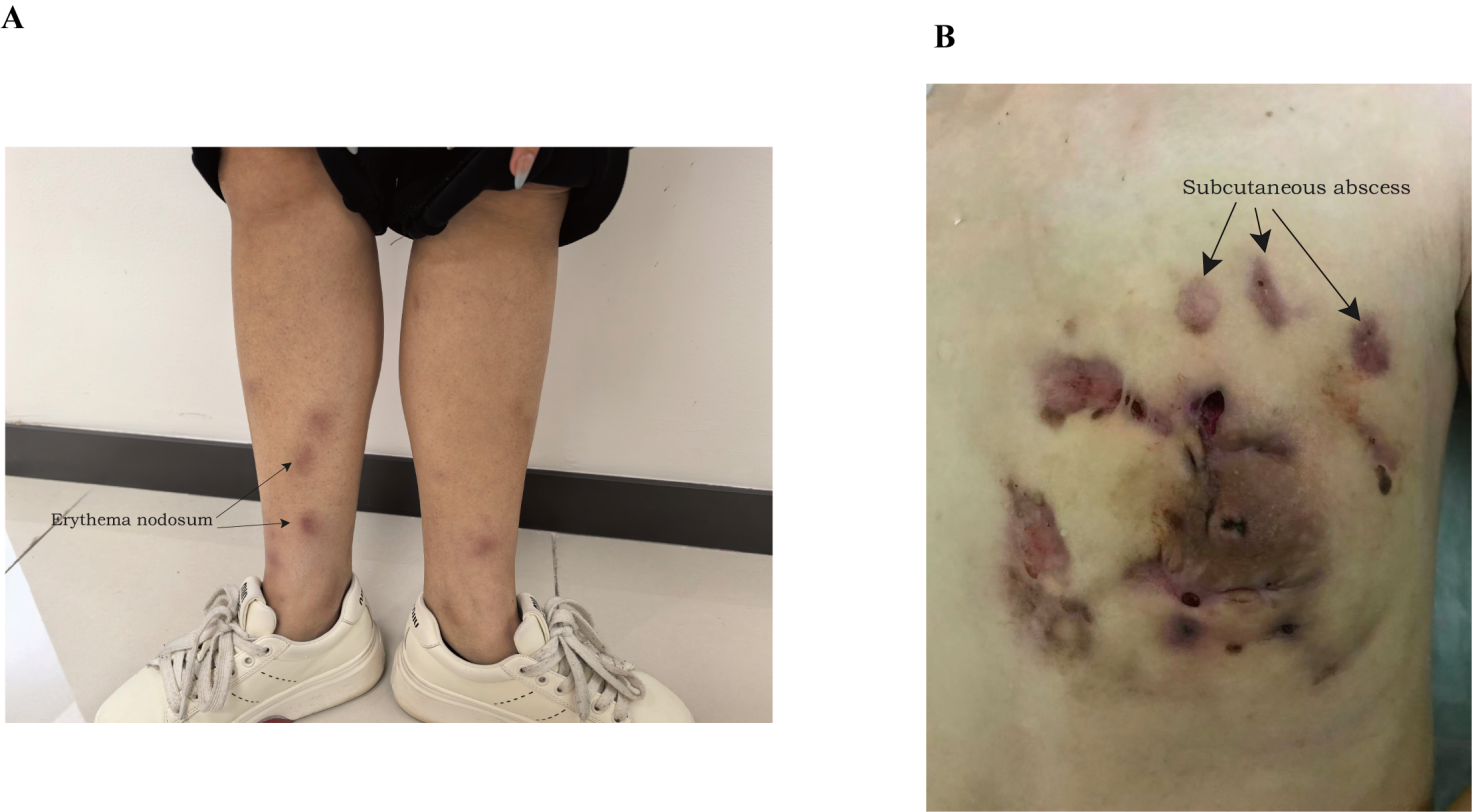
Clinical manifestations of GMENA syndrome. **(A)** Erythema nodosum on bilateral pretibial areas. **(B)** Subcutaneous abscess of the breast.

In recent years, accumulating evidence has shown that IGM can present with extramammary manifestations, most commonly erythema nodosum (EN), and less frequently arthritis. EN is a form of panniculitis characterized by painful subcutaneous erythematous nodules over the bilateral pretibial areas and is frequently associated with sarcoidosis, inflammatory bowel disease, infections, and autoimmune disorders (5) (**Figure 1A**). In 1987, Adams et al. first reported a case of IGM complicated by EN and arthritis (6). Since then, several case reports and case series have further described this clinical triad (7–9). In 2021, Parperis et al. systematically reviewed 29 patients and formally proposed the term “GMENA syndrome” (granulomatous mastitis, erythema nodosum, with or without arthritis), suggesting that it may represent an underrecognized systemic autoimmune disease (10).

However, a narrative review by Li et al. suggested that GMENA syndrome may represent a clinical entity with distinct immunological features. Most affected patients are women of childbearing age who frequently exhibit more intense systemic inflammation. Nevertheless, whether this phenotype indicates a more aggressive disease course or predicts different treatment outcomes remains unclear, as no large cohort study has provided definitive evidence to date (11). The current knowledge gap lies in the absence of systematic comparative analyses between IGM patients with and without GMENA syndrome in terms of demographic characteristics, disease severity, glucocorticoid sensitivity, and long-term recurrence rates. This limitation hampers clinicians’ ability to develop individualized and precise treatment strategies for this specific patient population.

Based on this, the present study conducted a single-center retrospective cohort analysis of 201 IGM patients, including 20 with GMENA syndrome (GMENA group) and 181 without (control group). We systematically compared the two groups in terms of demographic characteristics, disease severity, response to glucocorticoid therapy (including time to early improvement and total treatment duration), and recurrence rate, aiming to provide evidence-based support for the clinical recognition and individualized management of IGM with GMENA syndrome.

## 2 Methods

### 2.1 Study design and population

This was a single-center retrospective cohort study. We included all patients diagnosed with IGM at the Department of Breast and Thyroid Surgery, Third People’s Hospital of Chengdu, between January 2014 and January 2026. The diagnosis of IGM was based on a composite of the following criteria: (1) clinical presentation: palpable breast mass with or without pain, local skin erythema, abscess formation, or sinus tract development; (2) imaging findings: ultrasound or magnetic resonance imaging suggesting inflammatory lesions and excluding typical malignant features; and (3) pathological diagnosis (primary criterion): core needle biopsy or surgical excision biopsy confirming non-caseating granulomatous lobular mastitis, with the presence of multinucleated giant cells, epithelioid histiocytes, and lymphocytic infiltration, granulomas predominantly centered on the breast lobules, and negative acid-fast and periodic acid–Schiff (PAS) staining.

GMENA syndrome (granulomatous mastitis, erythema nodosum, with or without arthritis) is defined as a clinical constellation of IGM accompanied by erythema nodosum (EN), with or without arthritis. The diagnosis of EN was based on clinical presentation: the presence of subcutaneous painful, tender, erythematous nodules or plaques over the bilateral pretibial areas. In this study, patients who met the diagnostic criteria for IGM and presented with concurrent EN were assigned to the GMENA group.

Inclusion criteria: (1) age ≥ 18 years; (2) confirmed diagnosis of IGM; (3) complete baseline clinical data; (4) at least 3 months of follow-up, or a documented recurrence event within the first 3 months of follow-up.

Exclusion criteria: (1) concomitant malignancy; (2) active tuberculosis, fungal infection, or other specific infectious diseases; (3) a definite diagnosis of systemic autoimmune disease (e.g., systemic lupus erythematosus, rheumatoid arthritis, sarcoidosis, granulomatosis with polyangiitis); (4) missing key clinical or follow-up data.

In parallel with the diagnosis of IGM, we systematically excluded other conditions that may present with granulomatous inflammation, skin erythema, and arthritis. Infectious causes were ruled out by negative acid-fast and PAS staining, while systemic autoimmune and granulomatous disorders (including sarcoidosis, granulomatosis with polyangiitis, Behçet’s disease, and Crohn’s disease) were excluded based on the absence of characteristic clinical, imaging, and pathological features. In addition, all biopsies were reviewed to exclude cystic neutrophilic granulomatous mastitis (CNGM), defined by the presence of cystic spaces lined by neutrophils (12, 13). No cases met this criterion.

To ensure data completeness and minimize information bias over the extended study period, all electronic medical records and outpatient follow-up charts were systematically reviewed. Only patients with complete baseline data—including demographic characteristics, laboratory findings, imaging reports, treatment records, and follow-up information—were enrolled. Patients with missing key clinical variables or incomplete follow-up data were excluded from the analysis. Data extraction was performed independently by two investigators, with discrepancies resolved through consensus discussion. A total of 201 patients were ultimately enrolled. All patient data were obtained from the hospital’s electronic medical record system. The study protocol was approved by the Ethics Committee of the Third People’s Hospital of Chengdu (approval No. 2024-S-403), which granted a waiver of informed consent for this retrospective study.

### 2.2 Treatment protocol

For patients receiving oral prednisone, the starting dose was 0.75 mg/kg/day, which was determined based on the international consensus recommendation and our center’s previous experience (4, 14). This protocol remained unchanged throughout the study period. After achieving clinical improvement, the dose was tapered by 5–10 mg per week according to the patient’s response, and a maintenance dose of 2.5–5 mg/day was given once the disease was stable. If symptoms did not improve or worsened after 3 weeks of glucocorticoid therapy, discontinuation was considered (14).

All patients underwent routine blood count testing. If white blood cell count, neutrophil percentage, or C-reactive protein levels were elevated, antibiotics were added; if cultures were positive, antibiotics were administered according to susceptibility testing results. Antibiotics were used selectively based on infectious signs or culture results rather than as a routine component of the treatment protocol. No patient in this cohort received immunosuppressive agents such as methotrexate as initial therapy. Antibiotic regimens and durations were individualized based on clinical response and susceptibility testing results.

Surgical management: In this study, surgical management was categorized into two types. For patients with abscess formation, incision and drainage or ultrasound-guided aspiration was performed as a symptomatic treatment. For patients who showed no improvement or worsening after 3 weeks of glucocorticoid therapy, as well as those who experienced recurrence after glucocorticoid therapy, wide local excision was considered. Wide local excision was performed after the wound was free of purulent discharge or when a sinus tract had formed, and the local inflammation had subsided. This approach was chosen to minimize the risk of poor wound healing and to ensure optimal surgical outcomes in this cohort, in which the majority of patients were young women of childbearing age. It should be emphasized that we do not recommend wide local excision as a first-line treatment for GMENA syndrome, as extensive resection may lead to breast disfigurement and impair quality of life.

During treatment, patients were followed up in the outpatient clinic every two weeks to monitor disease status (through physical examination and ultrasound evaluation) and adverse drug reactions. After treatment completion, follow-up was conducted every three months either in the outpatient clinic or by telephone to assess prognosis and recurrence.

### 2.3 Data collection and variable definitions

The following data were retrospectively collected from the hospital’s electronic medical record system.

Demographic and clinical characteristics included: age (18–30, 31–40, ≥41 years); time from symptom onset to presentation (≤9, 10–20, 21–30, ≥31 days); laterality (left, right, or bilateral); history of breast trauma before onset (no/yes); smoking history (no/yes); and time since last breast feed (<3, 3–5, 6–10, ≥11 years, or nulliparous).

Lesion characteristics included: affected quadrant (upper inner quadrant, upper outer quadrant, lower inner quadrant, lower outer quadrant, central area, or multiple quadrants); maximum diameter of mass or abscess (≤3 cm or >3 cm); number of masses or abscesses (1 or >1); presence of subcutaneous abscess (no/yes) (**Figure 1B**); and presence of fever (no/yes).

Laboratory findings included: results of routine autoantibody panels (antinuclear antibody, anti-dsDNA antibody, anti-Sm antibody, rheumatoid factor, anti-SSA antibody, and anti-SSB antibody) reported as negative or positive, as well as pus culture results.

Time to early improvement after glucocorticoid initiation was categorized into four groups: <1 week, ≥1 week, no improvement, and declined treatment. The definition of early improvement varied by group. In the GMENA group, early improvement was defined as marked relief of joint pain, fading of erythema nodosum, or reduction in breast mass size (≥20% reduction in the maximum diameter of the breast mass measured by palpation or ultrasound). In the control group, early improvement was defined as reduction in breast mass size (≥20% reduction in the maximum diameter of the breast mass measured by palpation or ultrasound). No improvement was defined as lack of improvement or worsening of the above symptoms after 3 weeks of treatment.

Clinical improvement was defined as disappearance of pain and redness, with a ≥30% reduction in the maximum diameter of the breast mass (by palpation or ultrasound). In this study, clinical improvement was used as the indicator for initiating glucocorticoid tapering.

Clinical remission was defined as disappearance of pain and redness, with confirmation by both palpation and ultrasound of disappearance of mass, healing of abscess and sinus tract, and no active inflammation.

Treatment duration was defined as the time from first visit to achieving complete clinical remission, and was categorized as < 3 months, 3-6 months, or >6 months.

Recurrence was defined as reappearance of biopsy-confirmed or radiologically typical IGM lesions at the original site or in another quadrant after complete clinical remission.

### 2.4 Statistical analysis

Statistical analyses were performed using R version 4.3.0. Categorical variables were presented as frequencies (n) and percentages (%). Comparisons between the two groups were made using the Pearson chi-square test or Fisher’s exact test (when any expected cell count was <5). The standardized mean difference (SMD) and its 95% confidence interval (CI) were calculated to quantify the magnitude of differences between groups. An absolute SMD <0.2 was considered a negligible difference, 0.2–0.5 a small difference, 0.5–0.8 a moderate difference, and ≥0.8 a large difference. All tests were two-sided, and a *p*-value <0.05 was considered statistically significant.

## 3 Results

### 3.1 Baseline characteristics of patients

A total of 201 patients with idiopathic granulomatous mastitis (IGM) were enrolled in this study, including 20 (10.0%) with GMENA syndrome (GMENA group). Among these, 15 (75%) presented with joint pain, predominantly involving bilateral ankles. The remaining 181 patients (90.0%) without GMENA syndrome served as the control group. Baseline characteristics of the two groups are summarized in **Table 1**.

**Table 1:** Baseline characteristics of IGM patients in the GMENA and control group.

Characteristics	Categories	Overall (N = 201)	Control group (N = 181)	GMENA group (N = 20)	SMD (95% CI)	*P*-value
Age, n (%)	18-30	56 (27.9%)	52 (28.7%)	4 (20.0%)	0.84 (0.37, 1.31)	0.01^a^
	31-40	106 (52.7%)	90 (49.7%)	16 (80.0%)		
	≥41	39 (19.4%)	39 (21.5%)	0 (0.0%)		
Time from symptom onset to presentation (days), n (%)	≤9	60 (29.9%)	43 (23.8%)	17 (85.0%)	1.75 (1.26, 2.24)	<0.01^a^
	10-20	53 (26.4%)	50 (27.6%)	3 (15.0%)		
	21-30	51 (25.4%)	51 (28.2%)	0 (0.0%)		
	≥31	37 (18.4%)	37 (20.4%)	0 (0.0%)		
Lateral, n (%)	Left	104 (51.7%)	91 (50.3%)	13 (65.0%)	0.38 (-0.09, 0.84)	0.46^a^
	Right	91 (45.3%)	84 (46.4%)	7 (35.0%)		
	Bilateral	6 (3.0%)	6 (3.3%)	0 (0.0%)		
Trauma, n (%)	No	187 (93.0%)	168 (92.8%)	19 (95.0%)	0.09 (-0.37, 0.55)	>0.99^a^
	Yes	14 (7.0%)	13 (7.2%)	1 (5.0%)		
Smoking, n (%)	No	191 (95.0%)	173 (95.6%)	18 (90.0%)	0.22 (-0.25, 0.68)	0.26^a^
	Yes	10 (5.0%)	8 (4.4%)	2 (10.0%)		
Time since last breast feed (years), n (%)	<3	72 (35.8%)	64 (35.4%)	8 (40.0%)	0.71 (0.24, 1.18)	0.46^a^
	3-5	93 (46.3%)	81 (44.8%)	12 (60.0%)		
	6-10	17 (8.5%)	17 (9.4%)	0 (0.0%)		
	≥11	10 (5.0%)	10 (5.5%)	0 (0.0%)		
	Nulliparous	9 (4.5%)	9 (5.0%)	0 (0.0%)		
Affected quadrant, n (%)	Upper inner quadrant	31 (15.4%)	31 (17.1%)	0 (0.0%)	3.45 (2.88, 4.03)	<0.01^a^
	Upper outer quadrant	51 (25.4%)	51 (28.2%)	0 (0.0%)		
	Lower inner quadrant	28 (13.9%)	28 (15.5%)	0 (0.0%)		
	Lower outer quadrant	27 (13.4%)	27 (14.9%)	0 (0.0%)		
	Central area	18 (9.0%)	18 (9.9%)	0 (0.0%)		
	Multiple quadrants	46 (22.9%)	26 (14.4%)	20 (100.0%)		
Size of mass or abscess (cm), n (%)	≤3	47 (23.4%)	47 (26.0%)	0 (0.0%)	0.84 (0.37, 1.31)	<0.01^a^
	>3	154 (76.6%)	134 (74.0%)	20 (100.0%)		
Number of masses or abscesses, n (%)	1	147 (73.1%)	147 (81.2%)	0 (0.0%)	2.94 (2.40, 3.48)	<0.01^a^
	>1	54 (26.9%)	34 (18.8%)	20 (100.0%)		
Subcutaneous abscess, n (%)	No	161 (80.1%)	153 (84.5%)	8 (40.0%)	1.03 (0.56, 1.51)	<0.01^b^
	Yes	40 (19.9%)	28 (15.5%)	12 (60.0%)		
Fever, n (%)	No	174 (86.6%)	159 (87.8%)	15 (75.0%)	0.33 (-0.13, 0.80)	0.16^b^
	Yes	27 (13.4%)	22 (12.2%)	5 (25.0%)		

SMD, Standardized Mean Difference; CI, Confidence Interval.

^a^Fisher’s exact test.

^b^Pearson’s Chi-squared test.

Demographic characteristics and timing of presentation. There was a significant difference in age distribution between the GMENA and control groups (*p* = 0.01). Patients in the GMENA group were concentrated in the 31–40 years age bracket (80.0% vs. 49.7%), and no GMENA patient was aged ≥41 years (0% vs. 21.5%). Regarding time from symptom onset to presentation, the GMENA group presented significantly earlier than the control group (*p* < 0.01): 85.0% of GMENA patients presented within ≤9 days, compared with only 23.8% of controls.

Other clinical characteristics. No statistically significant differences were observed between the two groups in laterality, history of breast trauma before onset, smoking history, or time since last breast feed (all *p* > 0.05). Bilateral involvement was noted in 3.3% (6/181) of the control group and in 0% (0/20) of the GMENA group (*p* = 0.46).

Extent and severity of breast involvement. The GMENA group exhibited more extensive and severe disease. All GMENA patients (100.0%) were involved of multiple quadrants, compared with only 14.4% in the control group (*p* < 0.01). The maximum diameter of the mass or abscess was >3 cm in all GMENA patients (100.0%) versus 74.0% of controls (*p* < 0.01). Regarding the number of masses or abscesses, all GMENA patients had multifocal disease (100.0%), whereas only 18.8% of controls had multifocal disease (*p* < 0.01). Subcutaneous abscess was significantly more frequent in the GMENA group (60.0% vs. 15.5%, *p* < 0.01). Fever, as a systemic symptom, occurred in 25.0% of GMENA patients versus 12.2% of controls, but the difference did not reach statistical significance (*p* = 0.16).

### 3.2 Treatment response and prognosis

Time to early improvement after glucocorticoid initiation (**Table 2**). The GMENA group showed a significantly higher rate of early improvement than the control group (*p* < 0.01). In the GMENA group, 75.0% of patients achieved early improvement within 1 week, compared with 34.8% in the control group. The proportion of patients with no improvement with glucocorticoid therapy was 20.0% in the GMENA group versus 41.4% in the control group. In addition, 38 patients (21.0%) in the control group declined treatment, whereas no patient in the GMENA group refused therapy.

**Table 2:** Early improvement and prognosis of IGM patients in the GMENA and control group.

Characteristics	Categories	Overall (N = 201)	Control group (N = 181)	GMENA group (N = 20)	SMD (95% CI)	*P*-value
Time to early improvement after glucocorticoid initiation, n (%)	<1 week	78 (38.8%)	63 (34.8%)	15 (75.0%)	1.06 (0.59, 1.53)	<0.01
	≥1 week	6 (3.0%)	5 (2.8%)	1 (5.0%)		
	No response	79 (39.3%)	75 (41.4%)	4 (20.0%)		
	Declined treatment	38 (18.9%)	38 (21.0%)	0 (0.0%)		
Treatment duration (months), n (%)	<3	59 (29.4%)	59 (32.6%)	0 (0.0%)	1.65 (1.16,2.14)	<0.01
	3-6	113 (56.2%)	106 (58.6%)	7 (35.0%)		
	>6	29 (14.4%)	16 (8.8%)	13 (65.0%)		
Recurrence, n (%)	No	139 (69.2%)	120 (66.3%)	19 (95.0%)	0.78 (0.31,1.25)	<0.01
	Yes	62 (30.8%)	61 (33.7%)	1 (5.0%)		

SMD, Standardized Mean Difference; CI, Confidence Interval.

Fisher’s exact test.

Treatment duration. The GMENA group required a significantly longer treatment duration than the control group (*p* < 0.01). In the GMENA group, 65.0% of patients needed treatment for more than 6 months, compared with only 8.8% in the control group. Conversely, 32.6% of control patients had a treatment duration of less than 3 months, while no GMENA patient had such a short course.

Recurrence rate. The recurrence rate was significantly lower in the GMENA group than in the control group (*p* < 0.01). Only 1 patient (5.0%) in the GMENA group experienced recurrence, whereas the recurrence rate in the control group was 33.7%.

Use of antibiotics and surgical interventions: Antibiotic therapy was administered based on elevated inflammatory markers (white blood cell count, neutrophil percentage) or C-reactive protein levels. According to these criteria, 9 patients (45.0%) in the GMENA group and 88 patients (48.6%) in the control group received antibiotic treatment. For pus culture, 4 patients (20.0%) in the GMENA group and 41 patients (22.7%) in the control group yielded positive results, totaling 45 positive cultures. Bacterial culture was performed using lipid-enriched blood agar plates, and species identification was carried out using routine biochemical methods. Among the 45 positive cultures, 22 were identified as Corynebacterium kroppenstedtii (2 in the GMENA group and 20 in the control group); 15 were other Corynebacterium species, including C. amycolatum and C. minutissimum; and 8 were other non-Corynebacterium organisms.

Regarding surgical interventions, 20 patients (100.0%) in the GMENA group and 134 patients (74.0%) in the control group underwent incision and drainage or ultrasound-guided aspiration for abscess formation (including subcutaneous or deep abscesses). For wide local excision, no patient in the GMENA group underwent this procedure, while 11 patients (6.1%) in the control group underwent wide local excision.

### 3.3 Laboratory findings and skin manifestations

Regarding laboratory findings, routine autoantibody panels (including ANA, anti-dsDNA antibody, anti-Sm antibody, RF, anti-SSA antibody, and anti-SSB antibody) were negative in all 20 GMENA patients.

With respect to skin manifestations, erythema nodosum appeared 1–2 weeks after breast abscess formation in 19 GMENA patients (95.0%) and within 1 week after suppuration in 1 patient (5.0%). Of note, when erythema nodosum appeared, the amount of purulent discharge from the breast increased markedly; conversely, after effective prednisone treatment, the purulent discharge decreased substantially.

## 4 Discussion

In this single-center retrospective cohort study, we systematically compared 20 IGM patients with GMENA syndrome and 181 IGM patients without GMENA syndrome in terms of demographic characteristics, disease severity, response to glucocorticoid therapy, and prognosis. The results revealed a distinct clinical profile in the GMENA group characterized by “acute onset, high local inflammatory burden, rapid initial response to glucocorticoids but prolonged treatment duration, and low long-term recurrence rate,” whereas the control group exhibited a contrasting pattern of “relatively localized disease, delayed treatment response, short treatment duration but high recurrence rate.” These findings suggest that GMENA syndrome may represent a specific clinical subtype of IGM, driven by high-grade immune inflammation, and associated with a favorable prognosis after adequate treatment.

### 4.1 Clinical characteristics of GMENA syndrome: younger age, earlier presentation, more severe disease, and unilateral involvement

In this study, GMENA patients were concentrated in the 31–40 years age bracket (80.0% vs. 49.7% in controls), and none were aged ≥41 years (0% vs. 21.5%, *p* = 0.01), indicating that GMENA patients were younger at onset. Regarding timing of presentation, the GMENA group had a significantly shorter interval from symptom onset to presentation (85.0% vs. 23.8% presenting within ≤9 days). Several factors may explain this finding. First, the disease progressed more rapidly in the GMENA group, and 15 of 20 patients (75%) experienced ankle pain, prompting earlier medical consultation. Second, erythema nodosum, characterized by painful erythematous nodules or plaques over the bilateral pretibial areas, has obvious visible changes and pain, making it easily noticeable to patients and further reducing delay in seeking care.

In terms of lesion characteristics, the GMENA group exhibited significantly more severe breast involvement: all patients had multiquadrant involvement (100.0% vs. 14.4%), mass or abscess size >3 cm (100.0% vs. 74.0%), >1 mass or abscess (100.0% vs. 18.8%), and subcutaneous abscess (60.0% vs. 15.5%). These findings are largely consistent with those of Cetin et al., who reported multiquadrant involvement in 75% of patients with EN (15). Notably, all GMENA patients in our study had unilateral breast involvement, and none had bilateral disease. A narrative review by Li et al. summarized 45 reported GMENA cases up to 2025 and found that all had unilateral breast involvement, which is highly consistent with our results (11).

### 4.2 Glucocorticoid treatment response: rapid onset but prolonged treatment duration

Our study found that the GMENA group exhibited a pattern of “rapid onset of response but prolonged total treatment duration” to glucocorticoid therapy. In the GMENA group, 75.0% of patients achieved significant improvement within 1 week of treatment, compared with only 34.8% in the control group. This finding is consistent with the observation by Parperis et al., who reported that 86.2% of GMENA patients responded to glucocorticoid therapy (10). However, in stark contrast to the rapid response, the GMENA group required a significantly longer treatment duration: 65.0% of patients needed treatment for more than 6 months (vs. 8.8% in controls), and no patient achieved remission within 3 months (vs. 32.6% in controls). This “fast response, slow cure” pattern suggests that although GMENA patients have high initial sensitivity to glucocorticoids, their disease is more severe and requires a longer treatment course.

Possible reasons for this finding include the following. To exclude the possibility that incision and drainage itself contributed to the early improvement observed in the GMENA group, we performed a subgroup analysis comparing the early improvement rates between the two groups among patients who all underwent incision and drainage or ultrasound-guided aspiration. In this subgroup, the GMENA group still showed a significantly higher early improvement rate than the control group (75.0% vs. 35.1%, *p* < 0.001), indicating that the favorable corticosteroid response in GMENA syndrome remained the major driver of early improvement. Drainage alone could not explain the between-group difference. On the one hand, GMENA syndrome may involve a more intense immune-inflammatory response. Glucocorticoids can rapidly suppress acute inflammation (as reflected by resolution of EN and reduction in mass size), but the localized granulomatous inflammation in the breast may require a longer time to be completely eliminated. Premature tapering or discontinuation of glucocorticoids could lead to disease recurrence. Therefore, a prolonged course of low-dose glucocorticoid therapy is needed for GMENA syndrome, although the potential adverse effects of such therapy should not be overlooked. On the other hand, in some GMENA patients, the abscess wound may not have completely healed by the time glucocorticoids are discontinued, necessitating continued regular dressing changes until healing is achieved, which objectively extends the overall treatment duration.

Our findings differ markedly from those reported by Cetin et al., who observed a poor response to glucocorticoid therapy in patients with EN, with treatment failure (persistent or recurrent disease) occurring in 50% of cases, and consequently recommended surgery as first-line treatment (15). Several factors may account for this discrepancy. First, differences in treatment protocols: Cetin et al. used topical, oral, or combined glucocorticoid regimens, whereas our study predominantly employed oral prednisone, starting at 0.75 mg/kg/day, tapering by 5–10 mg per week after clinical response, and then maintaining a low dose of 2.5–5 mg/day – a strategy of “adequate initial dose, gradual tapering after response, and low-dose maintenance.” Second, ethnic differences: pharmacokinetics or receptor sensitivity to glucocorticoids may differ between Turkish and Chinese populations. Third, sample size: our GMENA group was larger (20 vs. 12 cases) and may better reflect the characteristics of the general patient population. In clinical practice, for patients with a poor response to glucocorticoids and severe joint pain, adding methotrexate achieved disease remission, although this strategy was not systematically applied in the present cohort. Given that most GMENA patients are young, extensive surgical resection could lead to breast disfigurement and seriously impair quality of life. Therefore, we reserve wide local excision for those who are unresponsive to or have recurrent disease despite optimal medical therapy, and only when absolutely necessary.

### 4.3 Recurrence rate: favorable long-term prognosis in the GMENA group

Despite having more severe disease and requiring longer treatment, the GMENA group had a significantly lower recurrence rate than the control group (5.0% vs. 33.7%, *p* < 0.01). The recurrence rates reported in the literature for GMENA syndrome are approximately 12% (3/25) in the review by Parperis et al. (10) and 13.3% (6/45) in the narrative review by Li et al. (11), both higher than the 5.0% observed in our study. Several factors may explain the low recurrence rate in our GMENA group. First, prolonged and adequate treatment (65% of patients received >6 months of therapy) ensured thorough elimination of local breast inflammation. Second, the visible improvement of erythema nodosum provided patients with tangible feedback on treatment efficacy, thereby enhancing adherence. Third, potential immunological mechanisms may be at play: intense initial immune activation could lead to rapid depletion of pathogenic immune cells, or trigger a self-limiting immunoregulatory circuit, resulting in a more stable remission state during long-term follow-up. Nonetheless, this is a single-center retrospective study, and given the rarity of the disease, only 20 patients were enrolled. Accumulation of more cases in the future will provide more robust evidence.

### 4.4 Immunological mechanisms of GMENA syndrome

The pathophysiology of GMENA syndrome remains unclear, but the clinical findings of this study provide important clues to its immunological nature. Unlike patients with classic IGM, those in the GMENA group exhibited multisystem involvement including EN, arthritis (75% presented with ankle pain), and a trend toward higher fever prevalence (25.0% vs. 12.2%). Routine autoantibody panels were negative in all GMENA patients, excluding classic systemic autoimmune diseases (e.g., SLE, RA) as the underlying cause. However, seronegativity does not negate immune mechanisms; several immune-mediated diseases such as Behçet’s syndrome and Sweet syndrome are also autoantibody-negative, involving innate immune dysregulation or T-cell-mediated responses rather than B-cell autoantibody production (16, 17). The acute onset, multisystem involvement, and rapid glucocorticoid response in GMENA suggest it may belong to the same immunological spectrum.

The paradoxical pattern of more severe disease but lower recurrence in the GMENA group suggests a unique immune regulatory profile. Although direct cytokine studies in GMENA are lacking, studies in classic IGM have reported elevated Th1/Th17-related cytokines (IL-8, IL-17, IL-22, IL-23) and Th1 infiltration with IFN-γ/TNF-α upregulation (18–24). By analogy, GMENA may involve hyperactivation of these pathways driving acute inflammation, coupled with effective negative feedback (e.g., regulatory T cells, IL-10) that reduces relapse after adequate therapy. Clinical evidence supporting systemic inflammation includes the appearance of EN (a cutaneous manifestation), ankle pain, and a temporal association: EN appeared 1-2 weeks after breast abscess formation in 95% of patients, and purulent discharge increased with EN then resolved in parallel with glucocorticoid treatment. This suggests that breast and systemic manifestations are two target organ expressions of the same underlying immune dysregulation, rather than a causal relationship.

### 4.5 Clinical implications and treatment recommendations

Based on the findings of this study, we propose the following recommendations for the clinical management of GMENA syndrome. First, early recognition is essential. In women of childbearing age with IGM, the presence of EN (painful erythematous nodules or plaques over the bilateral pretibial areas) should be routinely assessed. Once EN is confirmed, a diagnosis of GMENA syndrome should be made, and the patient should be informed that although the disease may be more severe, the response to glucocorticoid therapy is favorable. Second, prompt initiation of glucocorticoid therapy is recommended. GMENA patients respond rapidly to glucocorticoids, and the severity of the disease should not deter their use. In this study, 75% of patients achieved early improvement within one week, supporting glucocorticoids as first-line therapy for GMENA. Third, a prolonged consolidation phase is necessary. GMENA patients require a longer treatment duration (65% needed >6 months in this study). Clinicians should avoid premature tapering or discontinuation after symptom improvement and are advised to maintain therapy for some time after complete clinical remission to reduce the risk of recurrence. Fourth, unnecessary surgery should be avoided. The recurrence rate in our GMENA group was only 5.0%, much lower than the 50% treatment failure rate reported by Cetin et al. We do not support surgery, particularly mastectomy, as first-line treatment for GMENA. For patients with breast abscess formation, incision and drainage may be performed as symptomatic management, but wide local excision should be reserved only for those who fail to respond to or relapse after glucocorticoid therapy, and only when necessary.

## 5 Limitations

This study has several limitations. First, as a single-center retrospective study, it is subject to inherent selection bias and information bias, and causal inferences should be made with caution. Second, the sample size of the GMENA group was relatively small (20 patients). Although this represents one of the largest GMENA cohorts reported to date, the limited sample size may affect the statistical power of subgroup analyses and precluded multivariable regression analysis to adjust for potential confounding factors such as incision and drainage. In addition, the sample size disparity between the GMENA group and the control group was substantial; although we employed appropriate statistical methods (including Fisher’s exact test and standardized mean difference) to ensure the reliability of comparisons, the imbalance in sample size may still have some impact on the results. Third, treatment protocols were heterogeneous: some patients in the control group declined glucocorticoid therapy, while others discontinued treatment due to intolerance to side effects. Additionally, baseline disease severity varied across patients, and although we applied a standardized starting dose, subsequent dose tapering was individualized based on clinical response rather than initial severity. These factors may have influenced treatment response and recurrence rates. In addition, antibiotic regimens and durations were not standardized and were tailored to individual clinical conditions, which is inherent to the retrospective design of this study. Fourth, follow-up durations varied among patients, and long-term outcomes for some patients require further observation. Fifth, we did not measure serum cytokine levels, inflammatory markers, or autoantibody titers, which limits in-depth exploration of the immune mechanisms underlying GMENA.

## 6 Conclusion

This single-center retrospective cohort study systematically compared the clinical features, glucocorticoid treatment response, and prognosis of IGM patients with and without GMENA syndrome. The results demonstrate that GMENA syndrome is a distinct clinical subtype of IGM characterized by younger age, acute onset, severe breast involvement (multiquadrant, large mass, multifocal lesions, and a tendency to form subcutaneous abscesses), rapid response to glucocorticoid therapy, and, after an adequate prolonged consolidation course, favorable long-term outcomes with low recurrence. Clinicians should recognize GMENA syndrome early and adopt a treatment strategy of prompt initiation followed by sufficient consolidation, while avoiding unnecessary surgical intervention. Future multicenter prospective studies are needed to validate our findings and to explore the immunological mechanisms and biomarkers of GMENA syndrome.

## Data Availability

The original contributions presented in the study are included in the article/supplementary material. Further inquiries can be directed to the corresponding authors.
